# Enabling External Inquiries to an Existing Patient Registry by Using the Open Source Registry System for Rare Diseases: Demonstration of the System Using the European Society for Immunodeficiencies Registry

**DOI:** 10.2196/17420

**Published:** 2020-10-07

**Authors:** Raphael Scheible, Dennis Kadioglu, Stephan Ehl, Marco Blum, Martin Boeker, Michael Folz, Bodo Grimbacher, Jens Göbel, Christoph Klein, Alexandra Nieters, Stephan Rusch, Gerhard Kindle, Holger Storf

**Affiliations:** 1 Institute of Medical Biometry and Statistics Medical Center, Faculty of Medicine University of Freiburg Freiburg Germany; 2 Institute for Immunodeficiency Center for Chronic Immunodeficiency, Medical Center, Faculty of Medicine University of Freiburg Freiburg Germany; 3 Medical Informatics Group University Hospital Frankfurt Frankfurt am Main Germany; 4 German Center for Infection Research Satellite Center Freiburg Freiburg Germany; 5 Centre for Integrative Biological Signalling Studies University of Freiburg Freiburg Germany; 6 RESIST Cluster of Excellence 2155 to Hanover Medical School Satellite Center Freiburg Freiburg Germany; 7 Department of Pediatrics Dr von Hauner Children’s Hospital, University Hospital Ludwig Maximilians Universität München München Germany; 8 FREEZE Biobank, Center for Biobanking Medical Center, Faculty of Medicine University of Freiburg Freiburg Germany

**Keywords:** registry interoperability, collaboration in research, data findability, registry software

## Abstract

**Background:**

The German Network on Primary Immunodeficiency Diseases (PID-NET) utilizes the European Society for Immunodeficiencies (ESID) registry as a platform for collecting data. In the context of PID-NET data, we show how registries based on custom software can be made interoperable for better collaborative access to precollected data. The Open Source Registry System for Rare Diseases (*Open-Source-Registersystem für Seltene Erkrankungen* [OSSE], in German) provides patient organizations, physicians, scientists, and other parties with open source software for the creation of patient registries. In addition, the necessary interoperability between different registries based on the OSSE, as well as existing registries, is supported, which allows those registries to be confederated at both the national and international levels.

**Objective:**

Data from the PID-NET registry should be made available in an interoperable manner without losing data sovereignty by extending the existing custom software of the registry using the OSSE registry framework.

**Methods:**

This paper describes the following: (1) the installation and configuration of the OSSE bridgehead, (2) an approach using a free toolchain to set up the required interfaces to connect a registry with the OSSE bridgehead, and (3) the decentralized search, which allows the formulation of inquiries that are sent to a selected set of registries of interest.

**Results:**

PID-NET uses the established and highly customized ESID registry software. By setting up a so-called OSSE bridgehead, PID-NET data are made interoperable according to a federated approach, and centrally formulated inquiries for data can be received. As the first registry to use the OSSE bridgehead, the authors introduce an approach using a free toolchain to efficiently implement and maintain the required interfaces. Finally, to test and demonstrate the system, two inquiries are realized using the graphical query builder. By establishing and interconnecting an OSSE bridgehead with the underlying ESID registry, confederated queries for data can be received and, if desired, the inquirer can be contacted to further discuss any requirements for cooperation.

**Conclusions:**

The OSSE offers an infrastructure that provides the possibility of more collaborative and transparent research. The decentralized search functionality includes registries into one search application while still maintaining data sovereignty. The OSSE bridgehead enables any registry software to be integrated into the OSSE network. The proposed toolchain to set up the required interfaces consists of freely available software components that are well documented. The use of the decentralized search is uncomplicated to use and offers a well-structured, yet still improvable, graphical user interface to formulate queries.

## Introduction

### Background

The German Network on Primary Immunodeficiency Diseases (PID-NET) [1,2] was initiated as a research program of the Pediatric Immunology Working Group (*Arbeitsgemeinschaft Pädiatrische Immunologie* [API], in German) [3], funded by the German Ministry for Education and Research (*Bundesministerium für Bildung und Forschung* [BMBF], in German). The API brings together clinicians and scientists interested in clinical care and clinical research on patients with inborn errors of the immune system. After funding for the BMBF concluded, PID-NET remained a research network of the API, representing a collaborative platform to address various aspects of primary immunodeficiency (PID) research, from clinical care to basic science. For rare diseases, the number of patients on a regional scale is relatively low. However, on a larger scale, for example, countrywide or worldwide, patients with rare diseases are an important group in health care. According to Mahlaoui et al [4], the estimated minimal prevalence of PID in Europe is 11 per 100,000 inhabitants. As these patients and the health care experts specializing in their diseases are spread over several countries, rare diseases need structures for patient support and for disseminating scientific advances that differ from those for frequent diseases. On this basis, Germany published a national plan for rare diseases in 2013, which includes 52 policy proposals to guide and structure actions for treating rare diseases within the German health and social system [5]. In this context, the Open Source Registry System for Rare Diseases (*Open-Source-Registersystem für Seltene Erkrankungen* [OSSE], in German) project [6] was funded by the German Federal Ministry of Health. An important achievement of the OSSE was the generation of freely usable and easily adaptable software for rare disease registries. The software with additional information is publicly available [7]. The open source software can be used by patient organizations, physicians, scientists, and other parties for the creation of patient registries. As a result, the national registry landscape is empowered to comply with European principles regarding the establishment of minimum datasets and compliance with data quality standards; this is summarized in the European Union Committee of Experts on Rare Diseases recommendation on rare disease registries [8]. Also, the necessary interoperability between different registries is supported from the outset and allows those registries to be confederated. For this, the concept of decentralized searches was implemented, which complies with data protection requirements and preserves data sovereignty [9,10]. The OSSE concept focuses on the interoperability of registries and facilitates the process of establishing research networks at various levels (ie, regional and national), which is very attractive in the field of rare diseases. While OSSE-based registries can be interconnected directly, registries based on other software solutions, like the European Society for Immunodeficiencies (ESID) registry, are supported in participating by using the so-called *OSSE bridgehead*. In this work, the ESID registry is used as an example to demonstrate the process of extending a registry that was not initially built using the OSSE framework. By using the OSSE bridgehead, the functionality to receive decentralized search inquiries is added to the registry. The presented work describes the process of how to connect such a registry to the OSSE network and further suggests an approach that uses a free toolchain to implement and maintain the required interfaces.

### PID-NET and ESID Registry

Members of the interdisciplinary PID-NET consortium are working together to study inborn disorders of the innate and adaptive immune system. PID-NET focuses, in particular, on severe combined immunodeficiency diseases, autoimmune lymphoproliferative diseases, autoinflammatory diseases, and PID with colitis. One essential part of the PID-NET consortium is the registry in which data for analysis is stored. The registry was founded as part of the PID-NET consortium in 2009 and was funded by the BMBF until March 2018. The aim was to provide a tool to register PID patients for epidemiological and clinical research and to strengthen the network of PID researchers in Germany. Currently, more than 3000 patients from Germany are documented. When PID-NET decided to run a central register in 2009, the decision was made to use the existing ESID platform to document the German cases. The ESID registry uses custom software [11], which was completely re-engineered when the registry underwent a major redesign in 2014 [12].

### The OSSE Concept

Comprised of specialized modules, OSSE is a registry software toolkit with the goal of enabling scientists with a basic information technology background to build a registry for a specific rare disease. A form editor allows electronic case report forms to be defined for basic and longitudinal medical data. Furthermore, the back end derives the corresponding data schema from the structure of these forms. Each field or data element contained in these forms has to first be specified by metadata, including the data type, the measurement unit, and the value domain, among others, within a metadata repository (MDR) [13,14].

The integration of an MDR in the OSSE architecture facilitates the later process of integrating data from any registry, since all dataset specifications used in the respective registries can be retrieved from the MDR to ensure that third parties interpret data correctly.

**Figure 1:**
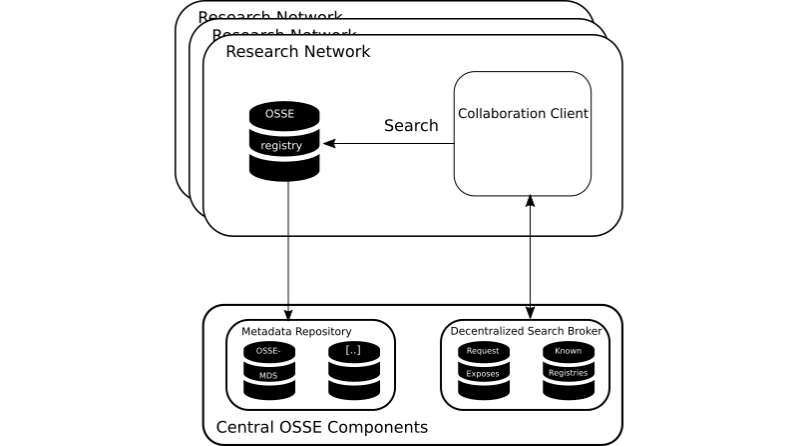
This illustration shows the different components of the OSSE and how they work together. Each registry, based on the OSSE metadata repository (MDS), models its data. The decentralized search broker publishes search inquiries to all selected known registries. Finally, the collaboration client of the selected registries searches for matching data. OSSE: Open Source Registry System for Rare Diseases (*Open-Source-Registersystem für Seltene Erkrankungen*, in German).

Furthermore, existing data elements can be reused when a new registry is established, and only additionally required items have to be newly defined. At the regional, national, and international levels, common dataset specifications can also be published in the MDR. The MDR component also allows for the retrieval of metadata items from other MDRs. Apart from self-defined value sets, the MDR provides access to standardized classifications, such as the ICD-10-GM (German Modification of the 10th revision of the International Statistical Classification of Diseases and Related Health Problems) or the ICD-O-3 (3rd edition of the International Classification of Diseases for Oncology) [6]. Figure 1 depicts the OSSE concept.

### The Distributed Search Principle

OSSE follows the principle that raw data, particularly of patients with rare diseases, should not leave the local registry. This is due to low acceptance among patients and data-owning scientists regarding the controllability of data collections and their usage by third parties, particularly outside the influence of national or Europe-wide data protection rules. Instead, OSSE provides a concept for a distributed search, which makes data from rare disease registries available, while respecting data sovereignty and privacy [9,15]. A central search broker allows for the definition of search queries based on the existing data elements in the MDR. Furthermore, the inquiring researcher has to include an abstract in the request describing the research question in detail.

The local request interface of each OSSE registry, called the *collaboration client*, downloads these queries and forwards them to the OSSE bridgehead, which then executes each query based on its local storage. If there is a nonempty result set, it is presented to the person in charge of each matching registry together with the exposé and the inquirer’s contact information. Finally, the data owner can review the result set and, if cooperation is desirable, contact the inquirer.

### Integration of Registries With the OSSE Bridgehead

Registries based on any non-OSSE software solution can be extended with the OSSE bridgehead to achieve the same interoperability. Hence, existing registries do not need to be converted to OSSE, which reduces barriers to collaboration. The OSSE bridgehead consists of the OSSE core components, including a local data storage component and the collaboration component. Data have to be imported from the registry by a periodical running process that can optionally access a local OSSE identifier management system based on the Mainzelliste [10,16], if the creation or modification of patient pseudonyms is required. To successfully import data into the bridgehead, each registry item has to be added as a data element defined in the MDR. The interface between the bridgehead and the existing registry uses XML following a specific XML Schema Definition (XSD). Based on metadata from the MDR and a data structure defined in a form editor, the XSD is automatically generated. The existing registry solution either has to implement an XML-based export interface or its native export format has to be transformed in a second step.

## Methods

### Installation and Configuration of the OSSE Bridgehead

The OSSE bridgehead runs on its own server to increase security by physically distinguishing the registry’s database from the local OSSE data storage. The bridgehead software is distributed as docker containers [17], which simplifies the installation and subsequent updates. After the installation, the bridgehead needs to be connected to at least one search broker. This is done by using an email verification process. After registration, one needs to specify the data elements and the data structure. In the MDR, all data elements of the registry’s dataset need to be defined by entering a definition, designation, and language. Further, the specification of each element’s data type is required. OSSE already offers data types, such as a list of permitted values, several numerical types for which ranges can be set, textual data, Boolean values, and types for date and time. Beyond that, for data elements using terminologies, a type catalog is created. These catalogs contain all the possible values. One can create new catalogs via an XML file upload.

Finally, represented by forms based on the MDR’s data elements, the data structure is created. Generally, two types of forms are provided: one for baseline data and one for longitudinal data. In our case, these forms contain data recorded at the first visit and at follow-ups, respectively. These forms are designed with the help of a user interface.

### Export Interface

Not every registry software offers a generic XML export, which is required by the OSSE bridgehead. With the help of the powerful, free, open source extract, transform, and load (ETL) tool, Pentaho [18,19], we suggest extending such registries by using an XML export. Further, based on an automatically generated XSD file downloaded from the OSSE bridgehead instance, which describes the dataset predefined in the MDR, an XSD transformation needs to be developed. This transformation translates data from the specialized XML export based on the registry’s model to a valid XML file that is compatible with the bridgehead.

After Pentaho is set up to perform the transformation jobs, the result needs to be pushed into the bridgehead, calling its representational state transfer (REST) interface. This ETL procedure can be periodically triggered by a continuous integration process, which is usually integrated into a modern version control system, such as GitLab [20]. This architecture offers flexibility regarding any code change and fast deployment.

### Querying OSSE Bridgeheads

The OSSE search broker enables the user to formulate search queries to connected registries. A centrally hosted user interface, for which an account is required, helps the user to create and manage queries. During the process of creating a new query, the submission of a proposal in PDF format is mandatory. Here, the user describes everything that could be relevant for the registry owner’s decision in the event of a collaboration. Further, the user has to assign a name to the query and optionally can add a project description. The inquiry will be addressed to a selection of registries of interest. The European Rare Disease Registry Infrastructure (ERDRI) [21] provides an ERDRI Directory of Registries (ERDRI.dor), which offers an overview of participating registries with additional descriptive information. Based on this information, ERDRI.dor offers a search that helps the user to find suitable registries for the inquiry. Finally, after formulating a query and submitting the web form, the search broker publishes the query, which is then downloaded by the collaboration clients of the selected registries of interest. Each one forwards the query to the OSSE bridgehead, which processes it and provides the result. If the result contains one or more matching patients, the registry operator can inspect the result set and the exposé in a user interface. Additionally, the platform allows for reprocessing of the query in order to update the result set based on the most recent data. The registry operator can choose to contact the inquirer to begin to negotiate the requirements for collaboration. The user who sent the query to the registries does not get a direct answer from the system. All communications regarding actual transfer of data have to be done externally (eg, via phone or email), beyond the scope of OSSE. Consequently, data never leave the registry server automatically.

## Results

### Configuration of the OSSE Bridgehead

As the documented PID-NET dataset has a wide range of parameters, we started the integration of the OSSE bridgehead with a selected subset of data elements.

In general, data elements that are used by different registries and, therefore, already exist as an entity in the MDR should be reused instead of entered redundantly. A search function helps users to find those elements. In case of minor differences regarding data element properties, data elements can be duplicated and subsequently adopted. An example is the existing *gender* data element with the properties *male* and *female*. The ESID registry further offers the option *unknown*. In this case, the already-entered *gender* data element could be used and extended by the missing property. In our case, the classification of PIDs is hierarchically structured in main categories and subcategories. In order to increase usability, we defined a custom catalog that represents the structure and is used to specify the respective data element for the PID classification. For *country of birth*, a catalog of a list of countries predefined by the system was chosen.

In particular, some items in the ESID registry are polymorphic (ie, data elements that may have two kinds of data types). For the item *date of last news*, originally one could assign a given date or a special value. If there is no news, the value *no_news* as type string is stored. OSSE does not support polymorphic data. In the MDR, the option *no_news* was encoded as the date 1850-01-01. For other data elements such as *date of documentation*, the type *date* is simply used. Items like *gene therapy* and *current route of administration* are realized with a list of permitted values.

We further distinguished between data recorded at the first visit and data collected during follow-up visits. This is required to achieve a semantically correct definition of our data when defining the two types of forms. In our case, these forms contain data recorded at the first visit and at follow-ups, respectively. These forms are designed with the help of a user interface. Multimedia Appendix 1 shows an excerpt of the PID-NET dataset.

### Export Interface

**Figure 2:**
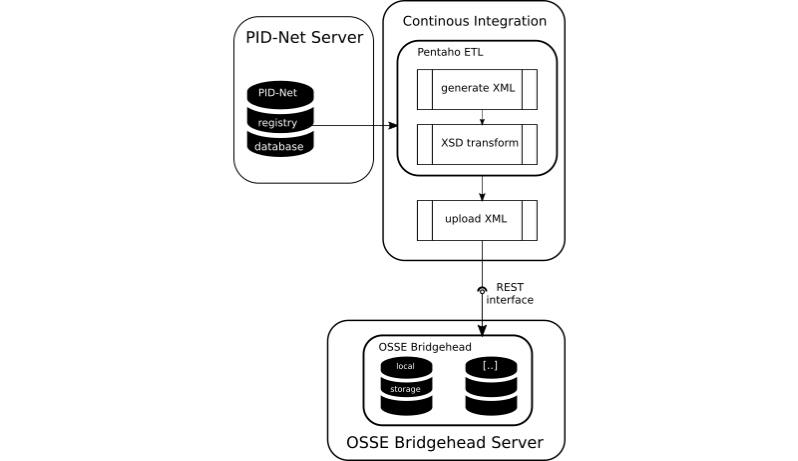
Connecting PID-NET via an OSSE bridgehead. The same structure could be used for any non-OSSE registry. ETL: extract, transform, and load; OSSE: Open Source Registry System for Rare Diseases (*Open-Source-Registersystem für Seltene Erkrankungen*, in German); PID-NET: German Network on Primary Immunodeficiency Diseases; REST: representational state transfer; XSD: XML Schema Definition.

Our registry software does not offer a generic XML export. With the help of an ETL process, we extended the ESID registry by using an XML export functionality. The ETL process further transforms the resulting XML into the bridgehead’s required format in order to periodically upload it to the OSSE bridgehead. The ETL process is triggered from within a continuous integration process in the version control software GitLab, which we installed on an internal server. In order to increase security, this server is safely located behind our corporate firewall and is only accessible from specific computers and by specific users. The continuous integration also uploads the export by using a shell script, which communicates with the bridgehead’s REST interface. Figure 2 illustrates the design of our implementation.

### Querying OSSE Bridgeheads

With the help of a publicly accessible web platform [22], queries to test the system were graphically created. Generally, a search query is constructed by logically connecting parameters and their required values. We modeled and performed the following research questions:

1. Which male patients have ever received hematopoietic stem cell transplantation (HSCT)?
2. Which patients have a common variable immunodeficiency (CVID) as their most recently documented PID diagnosis, or are still receiving immunoglobulin (Ig) replacements at initial registration or during follow-up?

As described earlier, we distinguished between first visit and follow-up data. This enables the user to create more specific queries (eg, formulating queries only considering follow-up data). Such a data design decision has to be made by the registry operator while modeling the data structure in the MDR and form editor.

Any query is performed by concatenating Boolean expressions. Therefore, the user needs to formulate research questions with logical conjunction and disjunction. The part of the second question “at initial registration or during follow-up” implies that the appropriate information refers to the data element at initial registration (init) as well as to the data element at follow-up (fu). Consequently, according to our data model, this requires both data elements to be requested. In order to show the logical expression of the questions, transferable to the visual query builder, we formulated it in an s-expression; this is related to the functional programming language Lisp [23,24] (see Textbox 1). As the second question is more complex, the user needs to nest the formulation of the expressions. Again, the s-expression is shown in Textbox 1.

**Formulations of s-expressions for the first and second questions.**

S-expression for the first query:

   (AND (EQUALS HSCT “YES”) (EQUALS SEX “M”))

S-expression for the second query:

   (AND

      (EQUALS PID_recent_, “CVID”)

      (OR

         (EQUALS Ig_fu_ “YES”)

         (EQUALS Ig_init_ “YES”)

      )

   )

**Figure 3:**
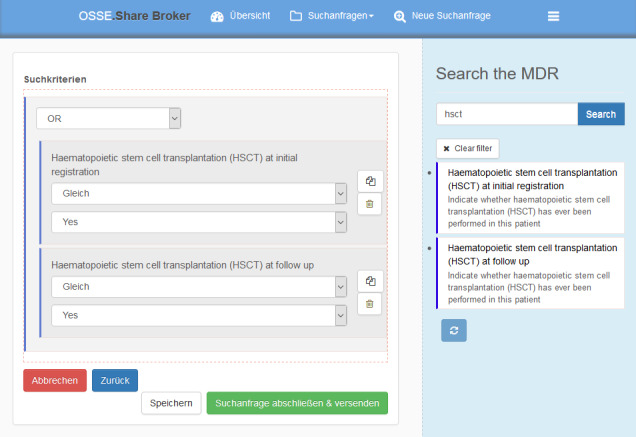
The graphical implementation of the first question, "Which male patients have ever received hematopoietic stem cell transplantation (HSCT)?" MDR: metadata repository.

**Figure 4:**
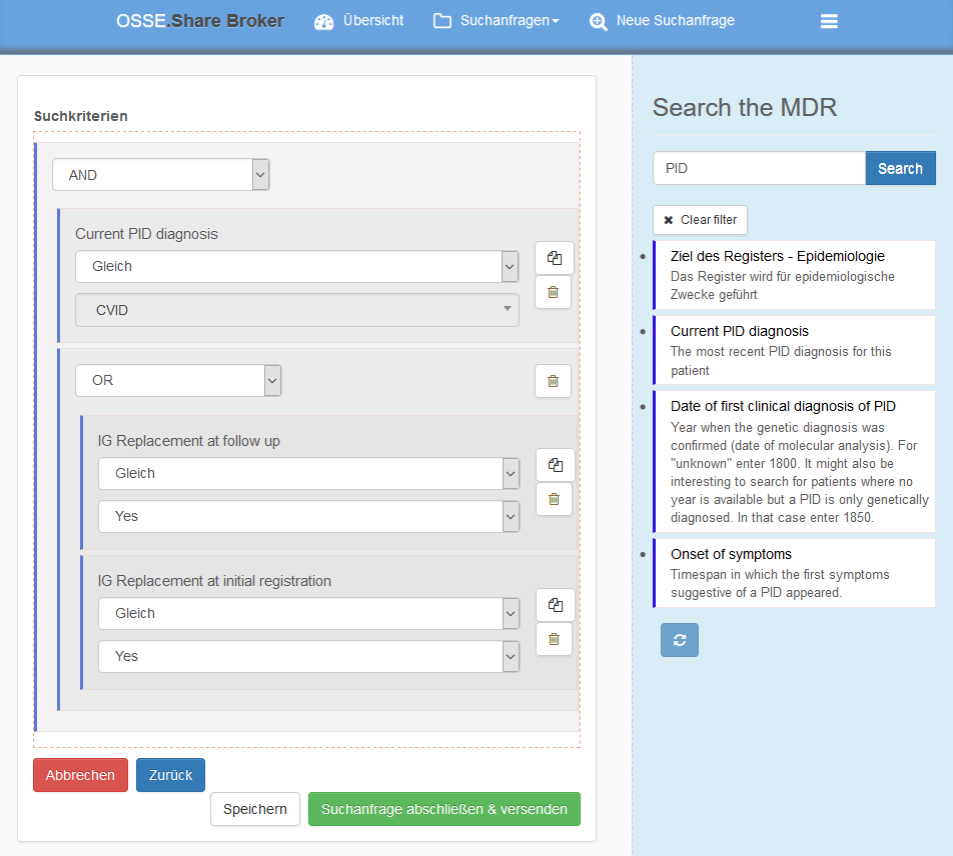
The graphical implementation of the second question requires nested constraints. The second question is "Which patients have a common variable immunodeficiency (CVID) as their most recently documented primary immunodeficiency (PID) diagnosis, or are still receiving immunoglobulin (Ig) replacements at initial registration or during follow-up?" MDR: metadata repository.

**Figure 5:**
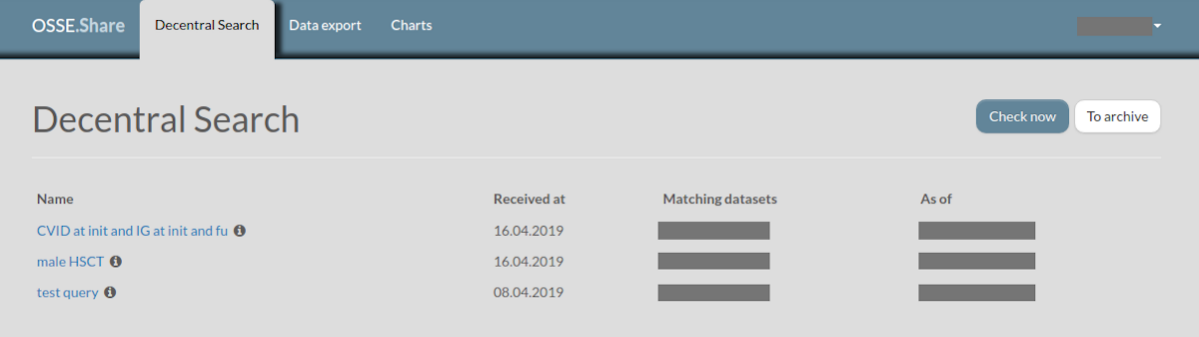
The local collaboration client, which is part of the OSSE bridgehead installation, lists all incoming inquiries. Further, one sees the date of last execution (As of) as well as the number of matching patients (Matching datasets). CVID: common variable immunodeficiency; fu: follow-up; HSCT: hematopoietic stem cell transplantation; IG: immunoglobulin; init: initial registration; OSSE: Open Source Registry System for Rare Diseases (*Open-Source-Registersystem für Seltene Erkrankungen*, in German).

Figure 3 shows the first expression translated into the graphical query builder. Figure 4 shows the second resulting query in the graphical query builder. After submitting a query, the local bridgehead for every registry of interest receives and locally executes the query. The local collaboration client of each bridgehead installation gives an overview of incoming inquiries and shows the number of matching patients in the bridgehead’s local storage (see Figure 5). Data always stay in the bridgehead’s local storage and, therefore, within the registry’s server infrastructure. Every desired exchange of data requires manual user actions as the system does not automatically generate responses that could reveal information about data. In the case of an incoming query, the data owner decides how to proceed. All subsequent communication regarding possible data transfer to the inquiring party needs to take place outside of the OSSE.

## Discussion

### Principal Findings

#### Overview

This work demonstrates how a registry that is not implemented with the OSSE registry framework can be made available in an interoperable manner without losing data sovereignty. With the help of a proposed toolchain, the ESID registry was successfully extended with required data export functionality in order to connect to a local installation of the OSSE bridgehead that adds the decentralized search functionality. Finally, the new feature was demonstrated by executing two example queries.

#### Limitations

The OSSE bridgehead is provided as a docker container working out of the box with minimal configuration. There is no need to compile and install multiple individual software components. Entering the dataset into the MDR and creating the forms is carried out by web-based user interfaces, which does not require special software, but does require a web browser. However, data harmonization could be fostered by proposing data element candidates: while creating a data element and typing in the designation, the system could search the MDR for similar pre-existing data elements to use. The most complicated development step was to program the ETL process, which extends PID-NET via an OSSE-compatible XML export and uploads this data into the bridgehead. Writing this process was necessary, as our registry has a complex data model and no generic XML export. We highly recommend the use of the Pentaho software. For registry software, which already includes a generic XML export, one could directly develop an Extensible Stylesheet Language (XSL) transformation in order to generate the final XML file. Further, we propose the implementation of a REST interface to the MDR and to extend the bridgehead with functionality to allow the development of a semiautomatic refresh of the MDR dataset and the forms of the bridgehead. There are data fields that will change over time, especially catalogs such as the PID classification. Currently, maintaining the system requires continuous manual work. The graphical query builder offers drag-and-drop and a search functionality. We suggest increasing the usability even further by limiting the selection of data elements. At the time of writing this paper, the column in which these elements are displayed shows every data element from all registries, instead of a limited selection of the registries of interest. Especially as the number of participating registries grows, limiting this list would have a huge impact on its usability and in terms of clarity.

The work presented here is a *proof of principle* for the technical feasibility to amend an existing registry by using external search functionalities. So far it has been evaluated by the PID experts operating the registry with typical queries from their experience.

#### Outlook

Following the idea of the recently developed ERDRI, we registered the ESID registry in the ERDRI.dor and uploaded an export of the specifications of our data elements (ie, the metadata) from OSSE.MDR into the ERDRI Metadata Repository (ERDRI.mdr). A stronger link between OSSE and ERDRI would be useful (ie, data in ERDRI.dor and ERDRI.mdr could be automatically updatable). Possible further steps would be to bring this work to a broader audience in the field of PID and encourage them to use this tool, rather than inquire with the registries themselves, and to include other registries and eventually other disease domains.

### Conclusions

In principle, the OSSE bridgehead allows registry software that is not created with the OSSE registry framework to be extended by a decentralized search functionality while maintaining data sovereignty. The only requirement is access to the raw data of the registry. This data access allows the registry to be extended by an ETL procedure that exports the data in the format the bridgehead requires. The decentralized search feature provides the possibility of more collaborative and transparent research. As the first registry to use the bridgehead, we successfully managed its integration into the OSSE network and successfully demonstrated the decentralized search functionality with two example queries. The setup of the OSSE bridgehead (ie, the installation, registration to a search broker, entering the dataset into the MDR, and creating the forms) could be further simplified. The most complex step was the implementation of the XML export interface, for which we suggest a specific and flexible free toolchain. Since this requires only a one-time effort, the expenditures are within justifiable limits.

Finally, we demonstrated that the OSSE can be used to interconnect registries, based on a federated search functionality, and ultimately made data from the ESID registry available in an interoperable manner without losing data sovereignty.

## Appendix

Multimedia Appendix 1 Excerpt of the dataset of PID-NET (German Network on Primary Immunodeficiency Diseases).

## Abbreviations

API: Pediatric Immunology Working Group (Arbeitsgemeinschaft Pädiatrische Immunologie, in German)
BMBF: German Ministry for Education and Research (Bundesministerium für Bildung und Forschung, in German)
CVID: common variable immunodeficiency
ERDRI: European Rare Disease Registry Infrastructure
ERDRI.dor: ERDRI Directory of Registries
ERDRI.mdr: ERDRI Metadata Repository
ESID: European Society for Immunodeficiencies
ETL: extract, transform, and load
fu: follow-up
HSCT: hematopoietic stem cell transplantation
ICD-10-GM: German Modification of the 10th revision of the International Statistical Classification of Diseases and Related Health Problems
ICD-O-3: 3rd edition of the International Classification of Diseases for Oncology
Ig: immunoglobulin
init: initial registration
MDR: metadata repository
OSSE: Open Source Registry System for Rare Diseases (*Open-Source-Registersystem für Seltene Erkrankungen*, in German)
PID: primary immunodeficiency
PID-NET: German Network on Primary Immunodeficiency Diseases
REST: representational state transfer
XSD: XML Schema Definition
XSL: Extensible Stylesheet Language
