# Supplementary material for: Enabling External Inquiries to an Existing Patient Registry by Using the Open Source Registry System for Rare Diseases: Demonstration of the System Using the European Society for Immunodeficiencies Registry
Source: JMIR Med Inform. 2020 Oct 7;8(10):e17420. doi: 10.2196/17420 (PMC7578818; doi:10.2196/17420)
Supplement: Multimedia Appendix 1 [file medinform_v8i10e17420_app1.pdf]

<table>
<tr><th>Designation</th><th>Definition</th><th>Baseline</th><th>Longitudinal</th><th>Possible values</th></tr>
<tr><td>Date of documentation</td><td>The date when the respective patient data was documented (generated based on the server clock)</td><td>yes</td><td>no</td><td>date</td></tr>
<tr><td>Year of birth</td><td>year of birth</td><td>yes</td><td>no</td><td>1850-2100</td></tr>
<tr><td>sex</td><td>sex</td><td>yes</td><td>no</td><td><table><tr><th>Label</th><th>Tooltip</th><th>Value</th></tr><tr><td>Male</td><td>Male</td><td>M</td></tr><tr><td>Female</td><td>Female</td><td>F</td></tr><tr><td>Unknown</td><td>Unknown</td><td>U</td></tr></table></td></tr>
<tr><td>Country of birth</td><td>Country in which the patient was born.</td><td>yes</td><td>yes</td><td>List of countries</td></tr>
<tr><td>IG replacement</td><td>Is the patient currently receiving Immunoglobulin replacement?</td><td>yes</td><td>no</td><td><table><tr><th>Label</th><th>Tooltip</th><th>Value</th></tr><tr><td>Yes</td><td>Yes</td><td>Y</td></tr><tr><td>No</td><td>No</td><td>N</td></tr><tr><td>Unknown</td><td>Unknown</td><td>U</td></tr></table></td></tr>
<tr><td>Current route of administration</td><td>Select the place of administration of IG-replacement. If the infusion is given in a hospital, indicate whether this is done during an inpatient or outpatient stay. If this is not known, choose the option ”hospital”. Only select this if IG-replacement is yes</td><td>no</td><td>no</td><td><table><tr><th>Label</th><th>Tooltip</th><th>Value</th></tr><tr><td>Home</td><td>Home</td><td>HOME</td></tr><tr><td>Hospital</td><td>Hospital</td><td>HOSP</td></tr><tr><td>Hospital: inpatient</td><td>Hospital: inpatient</td><td>HOSP</td></tr><tr><td>Hospital: outpatient</td><td>Hospital: outpatient</td><td>OUTPAT</td></tr><tr><td>Both</td><td>Home and hospital</td><td>BOTH</td></tr><tr><td>Office outpatient</td><td>Office outpatient</td><td>OFFICE_ OUTPAT</td></tr><tr><td>Unknown</td><td>Unknown</td><td>U</td></tr></table></td></tr>
<tr><td>Date of last news</td><td></td><td>no</td><td>yes</td><td>Date: yyyy-mm-dd or 1850-01-01 to indicate no_news</td></tr>
</table>

Table 1: Excerpt of the dataset of PID-NET. Data definitions that need to be entered into the MDR. Designation is the name of the data element in the query builder, whereas definition is displayed in the user interface as describing information.
